# Dermoscopic Clues of Histopathologically Aggressive Basal Cell Carcinoma Subtypes

**DOI:** 10.3390/medicina59020349

**Published:** 2023-02-13

**Authors:** Elisa Camela, Paula Ilut Anca, Konstantinos Lallas, Chryssoula Papageorgiou, Sofia-Magdalini Manoli, Theodosia Gkentsidi, Polychronia Eftychidou, Konstantinos Liopyris, Dimitrios Sgouros, Zoe Apalla, Aimilios Lallas

**Affiliations:** 1 Dermatology Unit, Department of Clinical Medicine and Surgery, University of Naples Federico II, 80131 Naples, Italy; 2 Department of Dermatology, “Iuliu Hațieganu” University of Medicine and Pharmacy, 400012 Cluj-Napoca, Romania; 3 Dermatology of Medical Oncology, School of Medicine, Faculty of Health Sciences, Aristotle University of Thessaloniki, 54453 Thessaloniki, Greece; 4 Second Dermatology Department, School of Medicine, Faculty of Health Sciences, Aristotle University of Thessaloniki, 54124 Thessaloniki, Greece; 5 First Dermatology Department, School of Medicine, Faculty of Health Sciences, Aristotle University of Thessaloniki, 54643 Thessaloniki, Greece; 6 Andreas Sygros Hospital for Cutaneous and Venereal Diseases, 16121 Athens, Greece; 7 Second Department of Dermatology and Venereology, ATTIKON General University Hospital, Medical School, National and Kapodistrian University, 12462 Athens, Greece

**Keywords:** basal cell carcinoma, BCC, high risk, subtypes, infiltrative, morpheaform, basosquamous, metatypical, micronodular, dermoscopy, predictors

## Abstract

*Background:* The group of histopathologically aggressive BCC subtypes includes morpheaform, micronodular, infiltrative and metatypical BCC. Since these tumors are at increased risk of recurring, micrographically controlled surgery is considered the best therapeutic option. Although dermoscopy significantly improves the clinical recognition of BCC, scarce evidence exists on their dermoscopic criteria. *Aim:* To investigate the dermoscopic characteristics of histopathologically aggressive BCC subtypes. *Materials and Methods:* Dermoscopic images of morpheaform, micronodular, infiltrative and metatypical BCC were analyzed for the presence of predefined variables. Descriptive and analytical statistics were performed. *Results:* Most histopathologically aggressive BCCs were located on the head and neck. Infiltrative was the most common subtype. All subtypes, except micronodular BCC, rarely displayed dermoscopic pigmentation. The most frequent dermoscopic features of infiltrative BCC were arborizing vessels (67.1%), shiny white structures (48.6%) and ulceration (52.9%). The features prevailing in morpheaform BCC were arborizing vessels (68.4%), ulceration (*n* = 12, 63.2%) and white porcelain areas (47.4%). Micronodular BCC was typified by milky red structureless areas (53.8%), arborizing vessels (53.8%), short fine telangiectasias (50%), ulceration (46.2%) and blue structures (57.7%). The most common findings in metatypical BCC were arborizing vessels (77.8%), shiny white structures (66.7%), ulceration (62.9%) and keratin mass (29.6%). *Limitations:* Study population of only white skin and relatively small sample size in some groups. *Conclusions:* Our study provided data on the clinical, dermoscopic and epidemiological characteristics of histopathologically aggressive BCCs.

## 1. Introduction

Basal cell carcinoma (BCC) represents the most common skin cancer all over the world, with a steadily increasing incidence. It is estimated that up to 2 million new BCCs are diagnosed annually in the United States [1]. In terms of prognosis, BCC has a very low metastatic potential but might be locally aggressive, invade surrounding and deep tissues, and cause significant disfigurement and morbidity.

The tendency to recur after surgical treatment significantly varies among different types of BCC. The most important determinant factor for the risk of recurrence is the histopathologic subtype. Accordingly, BCCs are classified into low-risk histopathologic subtypes (superficial and nodular) and high-risk ones (histopathologically aggressive BCCs).

The group of histopathologically aggressive BCC subtypes encompasses morpheaform, micronodular, infiltrative and metatypical BCC. The morpheaform subtype is characterized by thin cords of basaloid cells that are surrounded by a sclerotic collagenous stroma, mainly without peripheral palisading and stromal cleft formation [2]. In the micronodular variant, there are multiple small nests of basaloid cells in the dermis, with subtle peripheral palisading [2]. The infiltrative subtype is typified by thin cords with angulated ends of a few basaloid keratinocytes within mucinous stroma [2]. Lastly, metatypical BCC, otherwise referred to as basosquamous carcinoma, displays overlapping histologic features between BCC and squamous cell carcinoma (SCC) [2]; for this reason, there is some debate on whether it represents a true BCC subtype or a distinct entity [3].

Overall, the increased tendency of histopathologically aggressive BCCs to recur after surgery justifies a more careful surgical approach with micrographically controlled techniques or wider margins as compared to the low-risk subtypes, that can be adequately cured with conventional surgery with 3–4 mm safety margins, at least when located on non-critical anatomic sites [3,4,5].

Dermoscopy has an invaluable role in the diagnosis of BCC, with well-established criteria for superficial and nodular subtypes [6,7,8,9,10]. Because of the high diagnostic accuracy achieved with dermoscopy, diagnostic biopsies are not routinely required for superficial and small nodular BCCs, and the definite treatment can be directly applied, as also suggested by recent guidelines on BCC management. In contrast, diagnostic biopsies are considered optimal when a high-risk subtype is suspected because of the need to schedule a more demanding surgical approach, either with micrographic margin control or with wider surgical margins. However, the clinical recognition of high-risk BCC subtypes is often challenging, and scarce evidence exists on their dermoscopic criteria.

Hence, the aim of the present study is to investigate the dermoscopic criteria of the four histopathologically aggressive BCC subtypes and identify possible correlations with patients’ demographic characteristics and anatomic location.

## 2. Materials and Methods

A retrospective observational study was conducted at skin cancer referral centers in Greece between January 2018 and January 2022. The ethics committee approval was waived because the study was retrospective, did not affect in any way patients’ management and was conducted with appropriately anonymized datasets. The databases of our centers were screened for eligible patients, which were those with a definite histopathologic diagnosis of morpheaform, infiltrative, micronodular or metatypical BCC.

Inclusion criteria were the availability of a high-quality dermoscopic image and metadata, including age, sex and anatomic location. Tumors with mixed histopathologic subtypes (i.e., more than one subtype reported histopathologically) were excluded.

Patients’ age and sex, lesions’ location and the dermoscopic images were extracted from the database, anonymized and categorized into four groups according to the histopathologic diagnosis, namely morpheaform, infiltrative, micronodular and metatypical BCC. Subsequently, the dermoscopic images were randomized with the use of random numbers and provided for evaluation by two independent investigators with more than 5 years of experience in dermoscopy. The evaluators were blinded for the histopathologic diagnosis and were asked to score the presence or absence of predefined criteria.

The dermoscopic variables included in the analysis were selected based on pre-existing evidence and were the following: (i) type of vessels with seven variables scored as present or absent (arborizing, short fine telangiectasias, dotted/glomerular, hairpin, corkscrew, comma and polymorphic); (ii) white-colored structures with two variables scored as present or absent (shiny white structures and white porcelain areas); (iii) brown-colored structures with five variables scored as present or absent (maple leaf-like, spoke-wheel, concentric/dots, structureless and scattered brown dots). If at least one of these variables was scored as present, the evaluators were also asked to score the distribution with two possible values (up to 50% or >50% of the entire lesion); (iv) blue-colored structures with two variables scored as present or absent (globules and nests). If at least one of these variables was scored as present, the evaluators were also asked to score the distribution with two possible values (up to 50% or >50% of the entire lesion); (v) loss of substance with two variables scored as present or absent (multiple small erosions and ulceration); (vi) follicular structures with one variable scored as present or absent (plugs and/or rosettes); (vii) multiple aggregated yellow-white (MAY) globules (one variable scored as present or absent); (viii) white clods/milia-like structures (one variable scored as present or absent); (ix) keratin mass (one variable scored as present or absent); (x) superficial scales (one variable scored as present or absent); and (xi) milky red structureless areas (one variable scored as present or absent).

The dermoscopic images were captured with Dermlite Foto equipment (3Gen, Dana Point, CA, USA) at 10-fold magnification, using either polarized or nonpolarized light.

### Statistical Analysis

First, a descriptive analysis was conducted in order to calculate the mean and standard deviation of continuous variables and the frequencies of categorical variables. Following normality explorations, ANOVA Kruskal–Wallis was used to compare age and different BCC subtypes. In order to investigate possible associations between baseline characteristics (sex and location), dermoscopic criteria and different BCC subtypes, Pearson X^2^-test was used and univariate multinomial analysis was conducted. Infiltrative BCC was selected as the reference level for the comparisons since it was the most common subtype. All the statistical tests were two-sided and the level of significance was set at a = 0.05. The statistical package for social sciences statistical software (version 28.0, IBM SPSS Statistics for Windows, Armonk, NY, USA: IBM Corp) was used for data analysis.

## 3. Results

A total of 142 patients with high-risk BCC were included. Of them, 88 were males (62%), and 54 were females (48%), with an overall mean age at diagnosis of 70.2 ± 12.7 years. The demographic data of the patients of the study as well as the frequencies of the tumors’ baseline characteristics are illustrated in Table 1.

The distribution of the 142 tumors in the 4 study groups was as follows: morpheaform (*n* = 19, 13.4%), micronodular (*n* = 26, 18.3%), infiltrative (*n* = 70, 49.3%) and metatypical (*n* = 27, 19.0%). The most common location was the head and neck (*n* = 109, 76.8%), followed by the trunk in about 19% of cases, with very rare involvement of the limbs (4.2%).

Regarding dermoscopy, the most frequently observed criteria in the total sample were arborizing telangiectasias (*n* = 95, 66.9%), ulceration (*n* = 78, 54.9%), and shiny white structures (*n* = 67, 47.2%). Concerning pigmentation, the majority of BCCs did not display any pigment (*n* = 91, 64.1%). In those pigmented, blue structures prevailed over brown ones (30.3% versus 21.8%).

### 3.1. Frequencies per Group

The analytic results of the analysis per study group are shown in Table 1.

#### 3.1.1. Morpheaform BCCs

**Figure 1:**
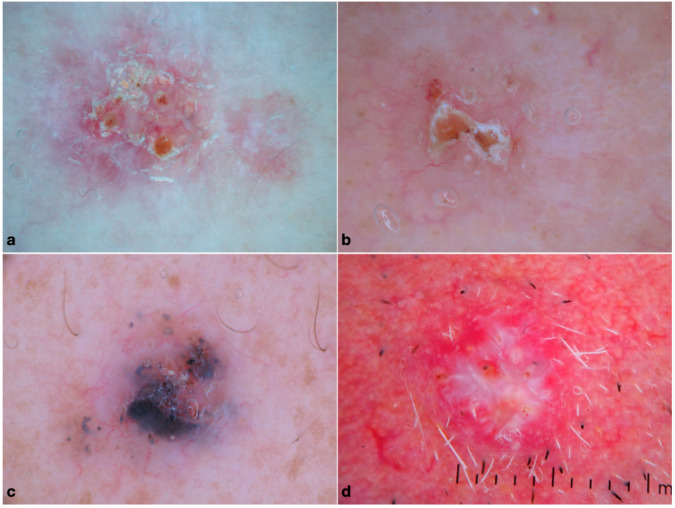
Dermoscopic pattern of the histopathologically aggressive BCCs: (**a**) infiltrative (shiny white structures, ulceration and arborizing vessels); (**b**) morpheaform (white clods, arborizing vessels, ulceration and white structureless areas); (**c**) micronodular (blue structures and arborizing vessels); and (**d**) metatypical (arborizing vessels, shiny white streaks, ulceration, white circles and plugs).

The sample of morpheaform BCCs included 19 patients (males [M] = 7, 36.8%) with a mean age at diagnosis of 69.4 ± 13 years. The most common location was the head and neck area (*n* = 15, 78.9%), followed by the trunk (*n* = 3, 15.8%). Concerning dermoscopy, most tumors lacked pigment (*n* = 16, 84.2%), and the most frequent features were arborizing telangiectasias (*n* = 13, 68.4%), ulceration (*n* = 12, 63.2%) and white porcelain areas (*n* = 9, 47.4%), as shown in Figure 1a.

#### 3.1.2. Micronodular BCCs

The group of micronodular BCCs included 26 patients (M = 14, 53.8%) with a mean age at diagnosis of 66.3 ± 17.6 years. Most of them developed on the head and neck region (*n* = 20, 76.9%), followed by the trunk (*n* = 5, 19.2%) and the limbs (*n* = 1, 3.8%). Dermoscopically, milky red structureless areas (*n* = 14, 53.8%), arborizing vessels and short fine telangiectasias (*n* = 14, 53.8% and *n* = 13, 50%, respectively), ulceration (*n* = 12, 46.2%) and blue structures (*n* = 15, 57.7%) were commonly observed (Figure 1b).

#### 3.1.3. Infiltrative BCCs

Infiltrative BCC was diagnosed in 70 patients (M = 45, 64.3%) with a mean age at diagnosis of 70.9 ± 11.2 years. Similarly to the previously discussed subtypes, the head and neck were the most common location in about 2/3 of cases, with 1/3 on the trunk and rare involvement of extremities. In dermoscopy, arborizing telangiectasias (*n* = 47, 67.1%), shiny white structures (*n* = 34, 48.6%) and ulceration (*n* = 37, 52.9%) were the most common features (Figure 1c).

#### 3.1.4. Metatypical BCCs

The group of metatypical BCCs included 27 patients (M = 22, 81.5%), with a mean age at diagnosis of 72.6 ± 10.3 years. The head and neck region represented the almost-exclusive location, accounting for 96.3% of cases. Arborizing telangiectasias (*n* = 21, 77.8%), shiny white structures (*n* = 18, 66.7%) and ulceration (*n* = 17, 62.9%) were the most frequently observed structures. Other common clues were follicular criteria and milky red structureless areas (40.7% each); of note, the percentage of BCC with keratin mass was higher than all other groups (*n* = 8, 29.6%). The dermoscopic pattern of metatypical BCC is shown in Figure 1d.

#### 3.1.5. Univariate Logistic Regression Analysis

Univariate logistic regression analysis was conducted to compare the likelihood of dermoscopic features according to the subtype, using infiltrative BCC as the reference group (Table 1 and Table 2). Overall, sex was statistically significantly associated with subtype (*p* < 0.01), whereas age and location were not (*p* > 0.05). Specifically, females had an increased probability of being affected by morpheaform BCCs (OR 3.08, 95% CI 1.07–8.84). The following dermoscopic criteria were found to be associated with the histopathologic subtype: follicular criteria (*p* = 0.012), white clods/milia-like cysts (*p* = 0.015), milky red structureless areas (*p* = 0.02), white porcelain areas (*p* = 0.01), blue globules/nests (*p* = 0.002) and brown-colored structures (*p* = 0.04). In detail, micronodular BCC displayed more frequently white clods/milia-like cysts (OR 6.07, [95% CI, 1.60–22.9]), milky red structureless areas (OR 3.13, [95% CI, 1.23–7.96]), blue globules/nests (OR 3.56, [95% CI, 1.30–9.70]) and > 50% of pigment (OR 12.93, [95% CI, 3.20–52.29]) when compared with infiltrative BCC. In contrast, the presence of white porcelain areas predicted infiltrative over micronodular BCC (OR of micronodular 0.16, [95% CI, 0.03–0.73]). Follicular criteria showed to be more frequently observed in metatypical as compared to infiltrative BCC (OR 4.66 [95% CI, 1.65–13.17]). No significant differences were found between infiltrative and morpheaform BCC in terms of dermoscopic features.

## 4. Discussion

Our study provides novel information on the clinical, epidemiological and dermoscopic characteristics of histopathologically aggressive BCC subtypes that might aid their clinical recognition and enhance appropriate management.

Overall, histopathologically aggressive BCCs were more frequent in males than females, with comparable age at diagnosis (70.1 versus 70.2 years). In general, the most frequently observed dermoscopic features in the whole sample were arborizing telangiectasias (*n* = 95, 66.9%), ulceration (*n* = 78, 54.9%), shiny white structures (*n* = 67, 47.2%) and absence of pigment (*n* = 91, 64.1%). The most common pigmented structures, when present, were the blue ones (*n* = 43, 30.3%), with globules prevailing over nests and over the combination of both (*n* = 29, 20.4%, versus *n* = 11, 7.7%, versus *n* = 3, 2.1%). These findings are in line with pre-existing evidence. In detail, Verduzco-Martine et al., in a previous study, suggested that the combined presence of ulceration and arborizing vessels was highly suggestive of high-risk BCCs, attributing to these features a highly predictive value [11]. This observation was further supported by the results of two other studies by Popadić et al. and Sgouros et al. that found ulceration to represent the strongest predictive factor of BCCs with a high risk of recurrence (increasing it up to 8-fold) [12,13]. Another feature that has been previously suggested to predict histopathologically aggressive BCC is the so-called MAY globules. This feature was recently introduced by Navarrete-Dechent et al. as a novel BCC-related criterion with high specificity (99.2%) and positive predictive value (95.3%) [14]. The latter study also included 32 histopathologically aggressive BCCs (infiltrative and morpheaform), and MAY globules were found in 18 (56.2%) of them, especially those located on the head and neck region [14]. In our study, MAY globules were seen in only 7% of histopathologically aggressive BCCs. The different sample sizes and the heterogeneity of the studied population may provide an explanation for the difference in the reported frequencies.

Analyzing each subgroup of our study separately, infiltrative BCC accounted for almost half of the included tumors, followed by micronodular and metatypical (around 20% each) and finally by morpheaform (less than 1/6 of cases). Our results, combined with previous evidence, allow for some conclusions on the dermoscopic morphology of each one of the four histopathologically aggressive BCC subtypes.

We found that infiltrative BCC is usually not pigmented and typified by the presence of arborizing vessels, shiny white structures and ulceration, in line with previous studies [15,16,17]. Interestingly, in a recent metanalysis by Reiter et al., the dermoscopic features of 288 infiltrative BCCs were analyzed, and the outcomes were the followings: arborizing vessels were present in 76% of the tumors (95% CI 59−77%), ulceration in 44% (95% CI 26−62%), and short fine telangiectasis in 40% of cases (95% CI 27−53%) [15]. Pigmentation was uncommon [16]. Leaf-like structures were rare findings (4%, 95% CI 0–10%) [16].

The dermoscopic morphology of metatypical BCC appears to be very similar to that of infiltrative BCC. Most tumors are non-pigmented (77.8%), and the most common dermoscopic features are arborizing vessels, shiny white structures and ulceration. Of note, keratin masses were highly observed in 29.6% of metatypical BCCs. These findings are in moderate consistency with the previous literature. In detail, our results are similar to those reported by Sgouros et al., with arborizing vessels being present in 88% of cases, ulceration/erosion in 72%, and superficial scales, keratin mass, shiny white structures in more than half (56%) [17]. Notably, the majority of the 22 metatypical carcinomas analyzed were non-pigmented (60%) in accordance with our data [16]. In contrast, Giacomel et al., in an equal number of metatypical carcinomas (*n* = 22), reported pigmentation as a common feature (blue-gray blotches [59.0%]); in addition, other frequent findings were: unfocused arborizing vessels, keratin mass and white structureless areas (73% each), superficial scales and ulceration (68.0%), white structures (64%) and blood spots in keratin mass (55%) [18]. Similarly, Akay et al. performed pattern analysis in 36 metatypical carcinomas, reporting that keratin mass (91.7%) was the most common dermoscopic feature, followed by superficial scaling (77.8%), white structureless areas (69.4%), white clods (66.7%) and blood spots on keratin mass (66.7%) [19]. The vascular pattern was mainly polymorphous (61.0%) [18]. This inconsistency in frequencies of dermoscopic features among different studies might be explained by the relatively small sample sizes, heterogeneity of terminology and different populations.

Morpheaform BCC was also usually non-pigmented (84.2%), ulcerated (63.2%) and displayed arborizing telangiectasias (68.4%), in line with pre-existing evidence [15,19]. In detail, in the systematic review conducted by Reiter et al. on the dermoscopic clues of BCC and its subtypes, 53 samples of morpheaform tumors were included and analyzed [16]. Overall, morpheaform BCCs were mainly non-pigmented, with one study reporting white porcelain areas as the prevailing dermoscopic feature (present in 75% of the sample) [15,20]. Interestingly, although rare, when present, pigmentation was commonly in the form of large blue-grey ovoid nests (13%, 95% CI 0−36%) [16].

In our study, white porcelain areas were present in almost half of the tumors (47.4%), more often than any other subtype. The latter feature is considered to correspond to the sclerotic stromal changes that are characteristic of this subtype.

Finally, micronodular BCC was more frequently pigmented as compared with the other three groups, with blue structures being present in 57.7% of the tumors. Other frequent dermoscopic features were milky red structureless areas, arborizing vessels, short fine telangiectasias and ulceration. A previous study reported similar findings with an even higher frequency of blue pigmentation, but it was conducted in a population with darker skin color [20]. Indeed, BCCs developing in individuals with dark phototypes (IV–VI, according to the Fitzpatrick classification) display pigment more frequently, regardless of the histological subtype, as compared with BCCs in patients with fair skin [20]. In a study by Vinay et al. on 19 micronodular BCCs in Indian patients, the prevailing structure was blue pigmentation, especially blue-white veil, being as high as in 84.2% of cases, followed by nests and dots (78.9% versus 68.4%) [20]. The highest frequency, as compared with our study, is probably explained by the different populations. However, the results of both studies converge to the conclusion that micronodular BCC often displays blue pigmentation, unlike the other three histopathologically aggressive subtypes. The latter finding is also clinically relevant because it has been demonstrated that pigment allows a better delineation of the tumor margins, facilitating surgical treatment [20].

Discriminating histopathologically aggressive BCCs from low-risk tumors would be of paramount clinical importance because it could guide the appropriate management. Analytically, the clinic-dermoscopic diagnosis of a histopathologically aggressive BCC should prompt confirmation with biopsy before designing a micrographically controlled surgery or excision with wide margins. In contrast, an accurate clinic-dermoscopic diagnosis of low-risk BCC would allow a direct excision in narrow safety margins without the need for a diagnostic biopsy. The current study is underpowered to answer this question because it did not include low-risk tumors. Based on the scarce pre-existing evidence, it seems that some of the features predominating in our series (ulceration and white shiny structures) might serve as predictors of histopathologically aggressive BCC [12,21]. However, this requires further elaboration in future studies that should involve large sample sizes and multiple readers in order to test also the interobserver agreement on dermoscopic structures. Up to then, existing evidence only allows the discrimination of superficial versus non-superficial BCC, which also has clinical relevance [8]. Interestingly, Sgouros et al., in a subanalysis comparing the dermoscopic features of histologically aggressive subtypes versus high-risk BCCs based on the current staging system, failed to find out significant differences between the two groups [12]. They concluded that factors, such as the tumor’s morphology and localization mirrored by dermoscopic pattern, may influence tumor behavior and clinical management other than histopathology [12]. Hence, dermoscopy may embody a precious tool to prevent unnecessary biopsies and guide the best therapeutic management.

The clinical differential diagnosis among high-risk BCC subtypes is of little relevance since the management does not differ substantially. However, we did perform a comparative analysis using infiltrative BCC as the reference category. The most interesting findings of the latter analysis were the observed differences between infiltrative and micronodular BCC. Specifically, micronodular BCC was more likely to display white clods/milia-like cysts, milky red structureless areas, blue globules/nests and pigment in more than 50% of the surface. In contrast, the presence of white porcelain areas was indicative of infiltrative BCC over micronodular. In addition, metatypical BCC seems to display follicular criteria more often as compared to the infiltrative subtype.

Our study has several limitations. First, it included only Caucasian patients, and therefore, the findings cannot be generalized to other populations. Second, the retrospective design is prone to recall and confirmation bias, which was addressed to some extent by the blinded evaluation. Third, although the total study sample size is relatively large, the number of cases in some groups might be underpowered to reveal significant differences. Finally, since the current study included only histopathologically aggressive BCCs, no conclusion can be extracted on the usefulness of the described criteria to discriminate histopathologically aggressive BCCs from low-risk subtypes.

## 5. Conclusions

The present study provides novel information on the dermoscopic morphology of histopathologically aggressive BCC subtypes that might facilitate their clinical recognition. Further research is needed to investigate the accuracy of subtype prediction with dermoscopy and determine whether it can be safely used to guide management.

## Figures and Tables

**Table 1:** Frequencies of demographic factors and dermoscopic criteria per histological diagnosis.

	Morpheaform *n* = 19, 13.4%	Micronodular *n* = 26, 18.3%	Infiltrative *n* = 70, 49.3%	Metatypical *n* = 27, 19.0%	x^2^-Test *p*-Value	Total *n* = 142
Age (mean ± SD)	69.4 ± 13.0	66.3 ± 17.6	70.9 ± 11.2	72.6 ± 10.3	0.44	70.2 ±12.7
Sex					**0.016**	
Male	7 (36.8)	14 (53.8)	45 (64.3)	22 (81.5)		88 (62)
Female	12 (63.2)	12 (46.2)	25 (35.7)	5 (18.5)		54 (38)
Location					0.18	
Head and neck	15 (78.9)	20 (76.9)	48 (68.6)	26 (96.3)		109 (76.8)
Trunk	3 (15.8)	5 (19.2)	19 (27.1)	0		27 (19)
Upper extremities	0	1 (3.8)	1 (1.4)	0		2 (1.4)
Lower extremities	1 (5.3)	0	2 (2.9)	1 (3.7)		4 (2.8)
*Non-pigmented structures*						
Follicular criteria	2 (10.5)	5 (19.2)	9 (12.9)	11 (40.7)	**0.012**	27 (19)
White clods/Milia-like structures	1 (5.3)	7 (26.9)	4 (5.7)	2 (7.4)	**0.015**	14 (9.9)
Multiple aggregated yellow-white (MAY) globules	3 (15.8)	3 (11.5)	3 (4.3)	1 (3.7)	0.23	10 (7.0)
Shiny white structures	6 (31.6)	9 (34.6)	34 (48.6)	18 (66.7)	0.053	67 (47.2)
Milky-red structureless areas	3 (15.8)	14 (53.8)	19 (27.1)	11 (40.7)	**0.024**	47 (33.1)
White porcelain areas	9 (47.4)	2 (7.7)	24 (34.3)	5 (18.5)	**0.010**	40 (28.2)
Superficial scales	4 (21.1)	3 (11.5)	13 (18.6)	4 (14.8)	0.80	24 (16.9)
Keratin mass	3 (15.8)	2 (7.7)	10 (14.3)	8 (29.6)	0.16	23 (16.2)
Vessels						
Arborizing telangiectasias	13 (68.4)	14 (53.8)	47 (67.1)	21 (77.8)	0.32	95 (66.9)
Short and superficial telangiectasias	5 (26.3)	13 (50)	25 (35.7)	9 (33.3)	0.38	52 (36.6)
Other vessels	1 (5.3)	3 (11.5)	16 (22.9)	4 (14.8)	0.23	24 (16.9)
Dotted/glomerular	0	1 (3.8)	6 (8.6)	2 (7.4)		9 (37.5)
Hairpin	1 (5.3)	1 (3.8)	6 (8.6)	1 (3.7)		9 (37.5)
Corkscrew	0	0	1 (1.4)	0		1 (4.2)
Multiple	0	1 (3.8)	3 (4.3)	1 (3.7)		5 (20.8)
Loss of substance						
Multiple small erosions	0	1 (3.8)	9 (12.9)	4 (14.8)	0.20	14 (9.9)
Ulceration	12 (63.2)	12 (46.2)	37 (52.9)	17 (62.9)	0.54	78 (54.9)
*Pigmented structures*						
Blue	2 (10.6)	15 (57.7)	21 (29.9)	5 (18.5)	**0.002**	43 (30.3)
Globules	1 (5.3)	12 (46.2)	15 (21.4)	1 (3.7)		29 (20.4)
Nests	1 (5.3)	1 (3.8)	5 (7.1)	4 (14.8)		11 (7.7)
Multiple	0	2 (7.7)	1 (1.4)	0		3 (2.1)
Brown	1 (5.3)	10 (38.5)	16 (22.9)	4 (18.4)	**0.044**	31 (21.8)
Leaf-like areas	0	1 (3.8)	1	0		2 (6.5)
Spoke-wheel areas	0	0	0	1 (3.7)		1 (3.2)
Concentric structures/dots	1 (5.3)	7 (26.9)	11 (15.7)	1 (3.7)		20 (14.1)
Structureless	0	4 (15.4)	5 (7.1)	2 (7.4)		11 (7.7)
Multiple	0	3 (11.5)	4 (5.7)	1 (3.7)		8 (5.6)
Scattered brown dots	1 (5.3)	2 (7.7)	4 (5.7)	0	0.56	6 (4.2)
Extension of pigment					**<0.001**	
Absent	16 (84.2)	8 (30.8)	46 (65.7)	21 (77.8)		91 (64.1)
<50%	3 (15.8)	9 (34.6)	20 (28.6)	3 (11.1)		35 (24.6)
>50%	0	9 (34.6)	4 (5.7)	3 (11.1)		16 (11.3)

Data are presented as n (%). SD, standard deviation, and MAY globules = multiple aggregated yellow-white globules. The *p*-value was considered significant if <0.05.

**Table 2:** Univariate multinomial analysis with dermoscopic predictors of different subtypes.

Reference Group: Infiltrative	Variables	OR	95% CI		*p*-Value
			Lower	Upper	
**Morpheaform vs. Infiltrative**	Sex (female)	3.086	1.077	8.840	0.036
**Micronodular vs. Infiltrative**	White clods/Milia-like structures	6.079	1.6074	22.990	0.008
	Milky red areas	3.132	1.231	7.967	0.017
	White porcelain areas	0.160	0.0348	0.734	0.018
	Blue globules	3.564	1.3085	9.706	0.013
	Pigment > 50%	12.937	3.2007	52.293	<0.001
**Metatypical vs. Infiltrative**	Follicular criteria	4.660	1.649	13.168	0.004

OR, odds ratio, and CI, confidence interval. The *p*-value was considered significant if <0.05.

## Data Availability

Further data will be available upon reasonable request.
